# Cortical candidates for self-other distinction based on visual and action cues: where do we stand?

**DOI:** 10.1007/s00429-025-03065-6

**Published:** 2026-01-07

**Authors:** Jakub Limanowski

**Affiliations:** Institut für Psychologie, Universität Greifswald, Franz-Mehring-Straße 47, 17489 Greifswald, Germany

**Keywords:** Action, Comparator model, Motor control, Self-other distinction, Vision

## Abstract

Distinguishing sensations that were generated by one’s actions (reafference) from those that were not may be grounded in the comparison of sensory and action cues; i.e., in evaluating sensory movement feedback against motor predictions by “comparator” modules in the brain. For the evaluation of visual movement feedback, brain imaging studies have converged on three candidate brain regions for such comparisons: the EBA, the STS, and the AG. Yet, the question whether the “action cues” received by those regions are motor or non-motor signals cannot conclusively be answered, partly due to the heterogeneity of the imaging results and differences in experimental methodology. Thus, there is an ongoing debate which of these regions implement visuomotor comparisons, rather than merely intersensory (e.g., visuoproprioceptive) comparisons blind to the cause of movement. In this mini review, I revisit the assumptions of classical (visuomotor) comparator models; discuss potential experimental biases resulting from non-motoric cues; highlight how differences in kinematic predictability result from different kinds of experimental visual feedback distortion—and how this could be capitalized on; and I discuss the potential promises and pitfalls of recent experimental approaches using adaptation designs and factorial visuomotor conflict designs with additional control over the locus of movement generation.

## Introduction

Perceiving myself as distinct from others includes distinguishing sensations generated by “me” (i.e., the sensory consequences of my actions) from those generated by external causes (e.g., other agents). This allows for the construction of adaptive models in the brain that use this distinction to guide and correct the organism’s action in a changing environment (Graziano and Botvinick 1999; Wolpert and Flanagan 2001; Jeannerod 2003; De Vignemont and Fourneret 2004; Gallese and Sinigaglia 2010). Pathologies, like in some forms of schizophrenia, hint at the profoundly negative consequences that a failure to distinguish between self- and other-generated sensations may have for embodied selfhood (Frith et al. 2000; Synofzik et al. 2010; Brown et al. 2013; Schmitter et al. 2025).

The difficulty of identifying self-generated sensations (e.g., for action control) is particularly evident in vision; i.e., in principle, seeing a body movement does not per se tell me whether it was caused by *my* action. Yet, already at 5 months of age, infants can discriminate between self-produced visual body movements and those produced by other infants (Bahrick and Watson 1985). A differential processing of matching vs. mismatching visual kinematics has been well documented in adults as well (e.g., Tsakiris et al. 2005; Brass et al. 2009; Salomon et al. 2013; Yon et al. 2018).

Computational architectures known as “comparator models” (see Section “Neurocomputational architectures for self-other distinction based on visual and action cues”) formulate how the comparison of sensory movement consequences with motor predictions in the brain underlies conscious self-other distinction and agency attribution (Blakemore et al. 1998; Gallagher 2000; Frith et al. 2000; Synofzik et al. 2008a; Haggard 2017). Of course, depending on its definition, the construction of a conscious “sense of agency” likely involves many more processes than sensorimotor comparisons, with different neuronal implementations (de Vignemont and Fourneret 2004; Synofzik et al. 2008a; Grünbaum and Christensen 2017; Wen 2019; Moore and Fletcher 2012). For instance, self-other distinction can also be attempted based on sensory data alone (i.e., inference about passive “body ownership” (Paillard 1999; Jeannerod 2003; Tsakiris 2010; Tsakiris et al. 2010). However, this inference is blind to the *cause* of the sensory data: Seeing a body movement does not per se tell me whether it is a consequence of *my* action I am seeing, or of someone else’s. This kind of self-other distinction can only be achieved by comparing “visual and action cues” (Jeannerod 2004). Thus, most of the above accounts agree on that the comparison of predicted and actual movement feedback (reafference) by the brain is one of the first and most important steps in establishing conscious self-other distinction. It is these comparisons—specifically, visuomotor comparisons—and their neurofunctional basis that are the focus of the present review.

Manipulations of visual movement feedback (i.e., its congruence with motor signals) belong to the most popular approaches to studying sensorimotor self-other distinction (Jeannerod 2003; Grünbaum and Christensen 2017; Wen 2019). Neuroimaging approaches to visuomotor self-other distinction have used various experimental manipulations of visual movement feedback, and the resulting brain activity increases have been interpreted in line with the comparator model; i.e., as indicating mismatch (error) processing as a potential first step in self-other distinction.

However, due to methodological differences and difficulties (see below), several key questions about visually based self-other distinction remain open, including where the “comparator module(s)” is (are) located in the brain and what kind of signals the visual input is compared against. Here, after a brief primer on the classical “comparator” model argument underlying sensorimotor self-other distinction in the brain, I shall revisit proposals converging on three key candidate areas for visuomotor comparisons in the brain, and briefly summarize the relevant key empirical findings. Then I shall review the most popular manipulations of visual movement feedback, focusing on their differences regarding the predictability of visual kinematics from motor signals, and what this implies for the experimental isolation of visuomotor comparisons from those based on non-motoric cues. Finally, I review human brain imaging studies that have combined manipulations of visual movement feedback with visuomotor adaptation designs, and with manipulations of the locus of movement generation (internal vs. external), and discuss what these approaches mean for isolating tentative visuomotor comparators. I shall end with a summary of the key open questions, alongside some suggestions as to how to proceed.

## Neurocomputational architectures for self-other distinction based on visual and action cues

**Fig. 1:**
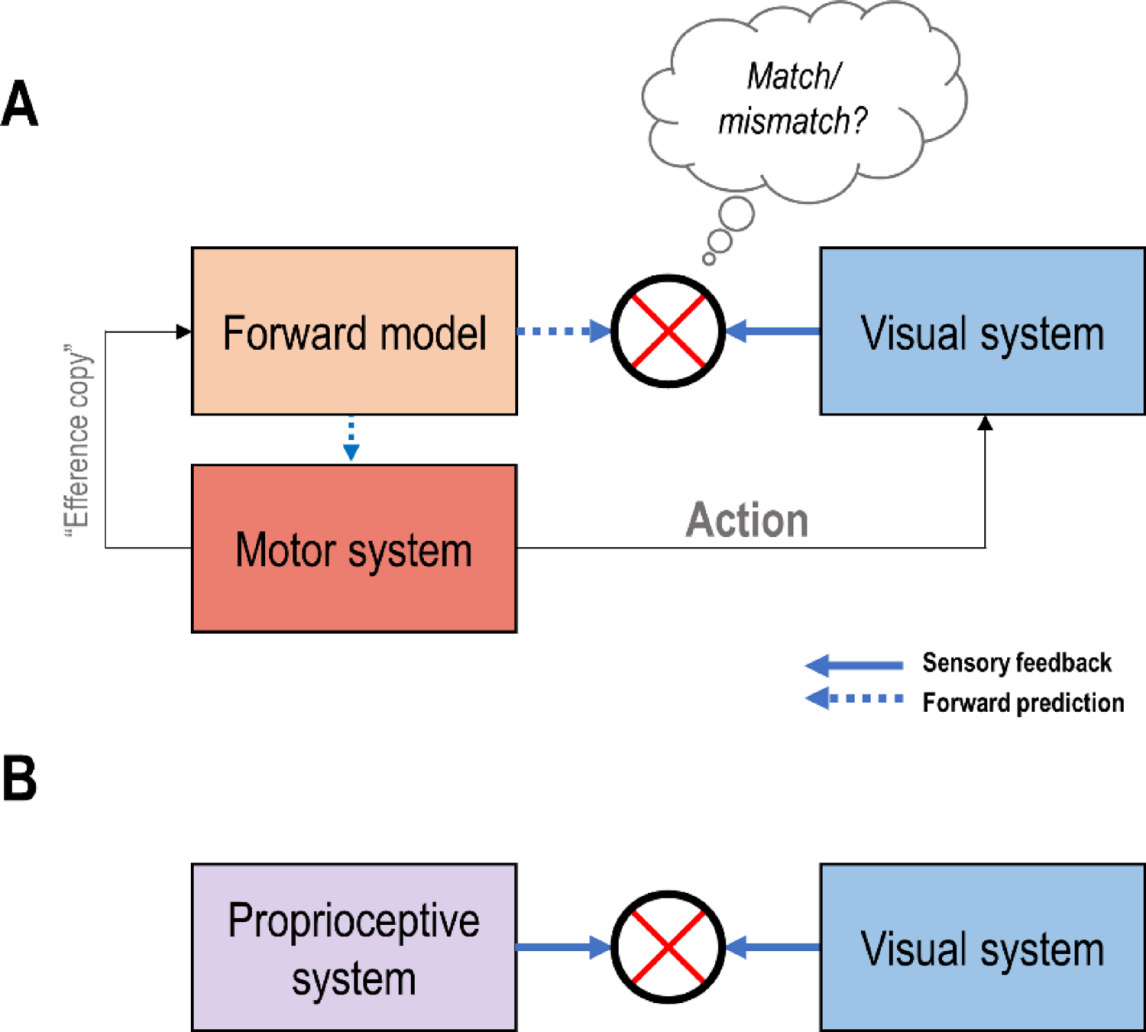
**A**: Simplified depiction of the “comparator model”. In parallel to generating action and the ensuing sensory (in this case, visual) movement feedback, the motor commands are fed as a copy to a “forward model” that uses them to predict the expected sensory (e.g., visual) movement consequences. These forward predictions can be used by the motor system for state estimation and “on-line” control instead of the actual sensory feedback, thus overcoming the problem of intrinsic conduction delays. Importantly, the sensory forward predictions can also be evaluated against the actual visual movement feedback (“reafference”) by a “comparator” module (crossed circle). Matches indicate correct predictions and, thus, likely self-generated movement consequences, which can, for instance, then be attenuated or “filtered out”. In contrast, mismatches mean that the observed movement was at least partly unpredicted, and therefore, likely generated externally (e.g., by someone else). This comparison fundamentally depends on the comparison of motor signals for action with sensory (re)afference. **B**: Another way to evaluate whether observed movements are of one’s own body. The seen limb position can be compared with its position conveyed by proprioceptors in the muscles and tendons (provided the appropriate transformations e.g. between the different coordinate systems used by vision and proprioception). Despite not receiving any motor signals, the detection of visuo-proprioceptive mismatches may also signal that the observed movement likely does not “belong” to one’s body, and can thus, in principle, also contribute to visually based self-other distinction of movement

Computational approaches to self-other distinction and its neurobiological implementation have largely been inspired by models from the motor control literature commonly referred to as “comparator models” (Blakemore et al. 1998; Gallagher 2000; Frith et al. 2000; Synofzik et al. 2008a). In short, these models rest upon the idea that, upon movement execution, the motor system sends a copy of the motor commands (Sperry 1950; von Holst and Mittelstaedt 1950; cf. Weiskrantz et al. 1971) to an internal “forward” model, which uses it to generate a prediction of the sensory consequences resulting from the execution of the intended (body) movement (Wolpert and Miall 1996; Desmurget and Grafton 2000; see Fig. 1A). These forward predictions can be used for state estimation and error correction instead of the actual sensory feedback, which reaches the brain only after substantial delays (Miall and Jackson 2006; Leib et al. 2024). This is a great benefit for sensory-guided action control (i.e., feedback control), because the correction of actions can now be based on anticipated sensory errors rather than having to wait for the actual sensory reafference.

Moreover, these predictions can be used to distinguish predicted reafference (i.e., the sensations generated by action) from unpredicted sensations (Wolpert and Flanagan 2001). Thus, a “comparator module” evaluates the actual, incoming sensory reafference against the (delayed) forward prediction issued by the motor system’s forward model (Fig. 1A). If this comparison results in a mismatch (prediction error), it means that the sensory feedback was unpredicted by one’s motor system and, therefore, likely not self-generated (Gallagher 2000; Frith et al. 2000; Jeannerod 2003; Synofzik et al. 2008a; Haggard 2017; Grünbaum and Christensen 2017). The predicted reafference can now be processed differently than unpredicted (exafferent) signals, which is important for contextually flexible motor control (see below).

The neurophysiological basis of motor/visuomotor control has been covered by several excellent reviews (e.g., Shadmehr et al. 2010; Wolpert and Miall 1996; Scott 2012; Adams et al. 2013; Archambault et al. 2015). While any single brain region could play a role in more than one kind of computation, there are several fitting candidates implementing specific computations based on their connectivity and known activity: The generation of motor plans and commands or predictions are likely implemented by premotor and primary motor areas, while forward models and state estimators may be implemented by the cerebellum and the posterior parietal cortex (Blakemore and Sirigu 2003; cf. Tzvi et al. 2022; Quirmbach and Limanowski 2024; Kilteni and Ehrsson 2024). These are likely supplemented by structures evaluating costs and rewards in, e.g., the basal ganglia (Scott 2012).

Most frameworks or hypotheses of self-other distinction assign “comparator” modules in the sense of Fig. 1A to the cerebral cortex. Thereby, a cortical visuomotor comparator should meet three criteria: Firstly, it should process visual movement feedback; i.e., it should receive afferents from earlier (primary or thalamic) visual areas and show activation by the observation of visual body movements. Secondly, it should receive forward predictions of visual movement feedback (see above) from the motor system; e.g., from the cerebellum, the PPC, or other (pre)motor cortices. Thirdly, the candidate brain area should respond differently to visual movement feedback that matches the forward predictions than to feedback that violates them.

Commonly, it is assumed that comparator modules in the sense of Fig. 1A signal mismatches (see above, e.g., Gallagher 2000; Frith et al. 2000; Haggard 2017). I.e., the prediction errors resulting from mismatching (i.e., unpredicted) sensory movement feedback are thought to be reflected by increased activity, e.g., increased hemodynamic responses in the comparator (e.g., Blakemore et al. 1998; Leube et al. 2003). This relates to the idea that self-generated sensory movement feedback is attenuated (e.g., Brown et al. 2013; Palmer et al. 2016; Kilteni and Ehrsson 2024). It should be noted, however, that self-generated (e.g., visual) feedback can also be augmented depending on task or control demands, which suggests more flexible, contextual gain control in the sensorimotor system (Scott et al. 2015; Azim and Seki 2019; Limanowski 2022). A possible reconciliation is offered by “sharpening” accounts suggesting increased neuronal activity, but only in a small proportion of neurons tuned to the self-generated sensory data (Yon et al. 2018; Reznik and Mukamel 2019). This could imply sequential, contextually weighted processing of expected and unexpected sensory information for optimal learning (Press et al. 2020). In this review, I follow the classical assumption that comparators can be identified through their increased responses to unpredicted sensations, although this assumption may need to be revisited in light of the above (Box 1). Other, complementary or subsequent processes such as the conscious self-attribution of action outcomes (“agency”) may be implemented by entirely different brain regions, such as the insula (Seghezzi et al. 2021; Sperduti et al. 2011; cf. Haggard 2017).

There is another key constraint on identifying visuomotor comparators, because, as noted, another kind of comparison of visual movement signals may also contribute to self-other distinction independently of motor signals and ensuing forward predictions: The seen limb position can be compared with its position conveyed by proprioceptors in the muscles and tendons—provided the nervous system has calculated the appropriate transformations between the different coordinate systems used by vision and proprioception (Fig. 1B, Weiskrantz et al. 1971; Makin et al. 2008; Tsakiris 2010; van Kemenade et al. 2019). In the simplest case, this means a momentary comparison of static visual and proprioceptive ‘snapshots’ of limb position, perhaps also informed by postural priors such as e.g. the “body schema” (specifying possible bodily states given the current configuration, Head and Holmes 1911; Paillard 1999; Bermúdez 2001; Synofzik et al. 2008b). Monkey neurophysiology and human imaging studies have identified neural correlates of such static visuo-proprioceptive comparisons in the posterior parietal cortex (PPC), the premotor cortex, the extrastriate visual cortex, and the cerebellum (Graziano and Botvinick 1999; cf. Ehrsson et al. 2004; Limanowski and Blankenburg 2016; Zimmermann et al. 2016). The detection of mismatches between predicted and sensed visual vs. proprioceptive limb posture may be a “non-motoric bodily cue” for self-other distinction, signaling that the observed movement likely does not “belong” to one’s body (Villa et al. 2022; cf. Bermúdez 2001; Jeannerod 2004). Crucially, however, this process is blind to the source of one’s body movements; and, as such, cannot identify whether an seen movement was self-*caused* or not (see above).

Naturally, sensory based and sensorimotor comparisons are tightly linked in determining body ownership and agency in most scenarios. A key challenge for imaging studies trying to locate ‘true’ visuomotor comparisons in the brain—according to the three criteria above—is to control for the neuronal correlates of such “non-motoric”, intersensory comparisons. Several studies have attempted to do this, identifying brain regions possibly implementing comparators in the sense of Fig. 1A.

## Possible loci of visuomotor comparisons in the brain

Visuomotor comparisons can, in principle, be calculated in all regions receiving visual and motor signals—but a few particularly fitting candidate regions have been identified. One of the first neurocognitive models for self-other distinction based on visual and action cues was proposed by Jeannerod (2004): In this model, the “extrastriate body area” (EBA, Downing et al. 2001) can distinguish self-produced body movements from those of others, whereby this information may then be used by the angular gyrus (AG) in the inferior PPC for the self- or other-attribution of action. Based on the signals from the EBA, the STS and premotor cortex could evaluate the social significance and intentions underlying the observed actions.

Jeannerod’s proposal drew upon the already well established role of the AG—more broadly, the inferior parietal lobule (IPL)—in the self- or other-attribution of action consequences (e.g., Sirigu et al. 1999; Farrer and Frith 2002; Farrer et al. 2003a, b). The AG, among other regions, has been associated with self-other distinction and the wider concept of the “sense of agency” by many studies (see reviews by Decety and Lamm 2007; David et al. 2008; Sperduti et al. 2011; Zito et al. 2020; Seghezzi et al. 2021). van Kemenade et al. (2017) have proposed a “supramodal” (domain general) comparator in the AG, which is supported by other recent findings of it showing multimodal action-outcome mismatch responses (Balslev et al. 2006; Ogawa et al. 2007; Krugwasser et al. 2019). This notion also aligns with the classification of the AG as a higher-level, multi-modal association cortex based on its extensive connectivity profile (cf. Passingham et al. 2002): AG connections include inputs from striatal, thalamic, and brainstem systems; short-range connections to parietal, occipital, and temporal lobes; and long-range connections to frontal association cortices (Petit et al. 2023; Yakar et al. 2023; Rockland 2023; Humphreys and Tibon 2023). Furthermore, AG activity covaries with premotor, parietal, and cerebellar activity, which could suggest functional connectivity with key sensorimotor control regions (Uddin et al. 2010; Mars et al., 2012).

Alternatively to supramodal regions like the AG, visuomotor comparisons could be implemented in specialized brain areas. Thus, a prominent hypothesis is that visuomotor comparisons should be implemented by brain areas processing visual motion—specifically, *visual biological motion*; i.e., that of living bodies (Jeannerod 2003, 2006; Kilner et al. 2007; Peelen and Downing, 2007; Friston et al. 2011). Models of biological motion have focused onto higher-order visual areas in the ventral stream; specifically, areas in the lateral occipitotemporal cortex (LOTC) including the EBA and hMT+, and inferior temporal regions in the STS (Perrett et al. 1985; Grossmann et al., 2000; Puce and Perrett 2003; Giese and Poggio 2003; Lange and Lappe 2006; Blake and Shiffrar, 2007).

Thus, a prominent model of biological motion perception (Giese and Poggio 2003) proposed two parallel processing streams, for form (processing sequences of visual “snapshots”, linked to ventral areas) and motion (processing optical flow, linked to dorsal areas), which converge on neurons in the STS. In this model, the EBA has been initially classified as part of the inferotemporal “form” pathway; i.e., processing visual “snapshots”. This resonates with another model by Lange and Lappe (2006), which proposed serial processing of form in the EBA and, subsequently, of global motion in the STS. The EBA is hence seen as an important part of the biological motion perception network, but associated primarily with the static analysis (of body posture) in sequential frames. Similarly, based on systematic comparisons of motion processing in the EBA vs. the STS, it has been argued that the EBA analyzes visual information of body posture in static snapshots (Vangeneugden et al. 2014; cf. Kontaris et al. 2009; Peelen et al. 2006). This would render it an unlikely visuomotor comparator. However, developments of the Giese and Poggio model have proposed interactions between both streams prior to the STS (Peuskens et al. 2005); and have aligned the EBA’s role more with that of the STS – locating it in the “motion” rather than the “form” pathway, with input from MT and other motion sensitive visual regions (Giese 2015). This aligns with findings suggesting that configurational and kinematic visual body motion information are integrated in the EBA (Jastorff and Orban 2009). In sum, while biological motion perception involves many more areas, including motion sensitive areas hMT + and the kinetic occipital area (Grossmann et al. 2000; Vaina et al. 2001), the STS and EBA are good candidates for processing visual biological motion in a way a potential “comparator” would need to.

However, the EBA and STS could also simply respond to visuo-proprioceptive mismatches (Fig. 1B); i.e., without evaluation against motor signals. Indeed, it is well documented that the EBA is sensitive to visuo-proprioceptive congruence even in the absence of movement; e.g., as demonstrated by studies on visuo-proprioceptive arm position integration (Ehrsson et al. 2004; Makin et al. 2008; Tsakiris 2010; Limanowski et al. 2014, 2016; Zimmermann et al. 2016). In contrast, the STS does not seem to respond to visuo-proprioceptive incongruence in the absence of movement (i.e., in static comparisons). This suggests any ‘mismatch’ response in the STS during mismatching visual movement feedback is likely unrelated to visuo-proprioceptive conflicts per se, and would be a strong argument for visuomotor (biological motion) comparisons in the sense of Fig. 1A.

Yet, a potential motor role of the EBA has also been suggested by the seminal findings of Astafiev and colleagues (2004), who demonstrated that the EBA was activated by unseen own body movements (see Limanowski and Blankenburg 2016; for a similar result). This was interpreted as potentially indicating the reception of motor signals such as an efference copy (e.g., Jeannerod 2004). The proposal of visuomotor self-other distinction in the EBA^1^ has since received quite some support—most notably, from studies associating EBA responses with the processing of mismatching visual movement feedback (e.g., David et al. 2007, 2008; Yomogida et al. 2010; Limanowski et al. 2017; see David et al. 2008; Zito et al. 2020; for reviews). But it has also been challenged (as discussed in e.g. the reviews by Peelen and Downing 2007; Lingnau and Downing 2015), with critics insisting on the functional definition of the EBA as a classical category-specific ventral stream region based on its significant preference for visual stimuli that depict human body parts (Downing et al. 2001). Thus, there is an ongoing debate about whether the EBA processes dynamic or static movement signals (see above); and whether the information it possibly receives about own body movement, beyond vision, is of motor (i.e., corollary discharge) or sensory (i.e., proprioceptive) nature. As the EBA is a functionally, not cytoarchitectonically defined region (Lingnau and Downing 2015), the main insights about its connectivity stem from functional or effective connectivity studies. These studies have shown correlated activity of the EBA and the PPC (Limanowski et al., 2014, 2017; Zimmermann et al. 2018; Moayedi et al. 2021). Zimmermann et al. (2018) found that, functionally and structurally, the EBA was more strongly connected to dorsal stream regions than other category-selective ventral stream regions. Thus, its connectivity profile, in principle, aligns with a potential motor role of the EBA (see above and Zimmermann et al. 2016).

The STS is the most consistently emphasized region in models of biological motion perception (see above). This region shows strong responses during action observation, and has been associated with social perception from visual cues, including imitation from observation (Allison et al. 2000; Iacoboni et al. 2001). Recent models even suggest it as a core node in a possible third visual stream, in between the classical ventral and dorsal ones, which underlies the unique social abilities of humans (Pitcher and Ungerleider 2021; Patel et al. 2019). Notably, the STS’s strong responses during passive action observation can also be interpreted as indicating self-other distinction based on the comparison of one’s (absent or contrary) motor signals with the seen action (Leube et al. 2003; Kilner et al. 2007; cf. Frith and Frith 1999; Miall 2003). In line with this idea, several human brain imaging studies have found increased responses in the STS, not in the EBA (nor AG), to mismatching visual movement feedback (Leube et al. 2003; Kontaris et al. 2009; Limanowski et al. 2018). As in case of the EBA (see above), a common interpretation of the STS is as a non-motor region; i.e., as lacking motor properties, in contrast to other, parieto-frontal regions involved in action observation and understanding (Rizzolatti and Sinigaglia 2010). The STS may receive afferents from the ventral stream areas (i.e., from the EBA/hMT + and other temporal regions; cf. Giese and Poggio 2003; Dasgupta et al. 2017; Patel et al. 2019), but also from the PPC (Yeterian and Pandya 1991; Seltzer et al. 1989, in monkeys); while it projects to frontal (premotor) areas (e.g., Luppino et al. 2001). As for the EBA (Zimmermann et al. 2018; see above), a meta-analysis showed that the posterior STS coactivated more strongly with dorsal stream regions; i.e., the PPC and pre- and supplementary motor areas (Erickson et al. 2017). Likewise, some studies reported STS activation by movements without vision (Molenberghs et al. 2010; Limanowski et al. 2018).

**Fig. 2:**
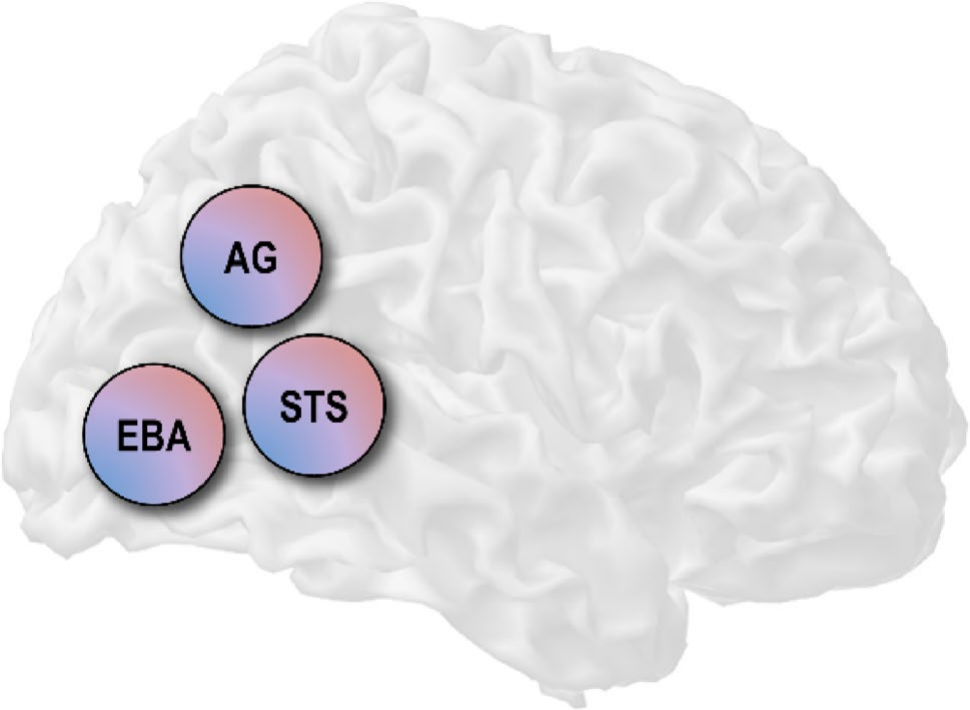
Three candidate regions for the implementation of self-other distinction of body movements based on visual and action cues. All three regions are activated by action observation, show elevated responses to distorted visual movement feedback, and potentially receive motor signals. *EBA* Extrastriate body area; *STS* Superior temporal sulcus; *AG* Angular gyrus

In sum, of the regions proposed in Jeannerod’s model, the AG, the EBA, but also the STS are promising potential candidates for visually based self-other distinction of action (Fig. 2). Firstly, all three regions are activated by action observation (see Caspers et al. 2010; for a meta-analysis); i.e., they process visual body movements. Secondly, a recent meta-analysis of human fMRI studies (Zito et al. 2020; cf. also Sperduti et al. 2011) identified hemodynamic correlates of “negative agency” in visuomotor tasks confined to essentially only these three brain areas (AG/inferior PPC, STS, and occipitotemporal clusters potentially encompassing the EBA). This aligns with the assumed sensitivity of these regions to visuomotor mismatches. However, only few published imaging studies used designs suited to isolate visuomotor from visuo-proprioceptive comparisons (see below). Thus, the key question remains: Which of these brain areas, if any, use motor afferents to ascribe visual movement feedback to oneself or others.

Importantly, all three candidate areas could, potentially, receive motor signals based on their known functional and structural connectivity: The motor role of the PPC is undisputed (Wolpert et al. 1998; Andersen and Buneo 2002), and the anatomical and functional connectivity of the AG suggests several possibilities of receiving motor signals from e.g. parietal, premotor, or potentially even cerebellar areas (Mars et al., 2012; Uddin et al. 2010; see above). However, although there is some evidence for at least functional coupling between the EBA/STS and motor regions like the premotor or parietal cortex (e.g., Patel et al. 2019; see above), it is still an open question whether the EBA and the STS receive motor signals e.g. from regions implementing forward models (Box 1). 

Therefore, the exact function of the AG, EBA, and STS in visuomotor self-other distinction is still not fully clear. Besides open questions about network activity/connectivity, this is predominantly due to the heterogeneity of the published functional results (see van Kemenade et al. 2019; for a discussion). This heterogeneity may stem from differences in experimental design and methodology, as discussed in the following.

## Experimentally targeting visuomotor comparisons

### Manipulations of visuomotor congruence and why their differences matter

**Fig. 3:**
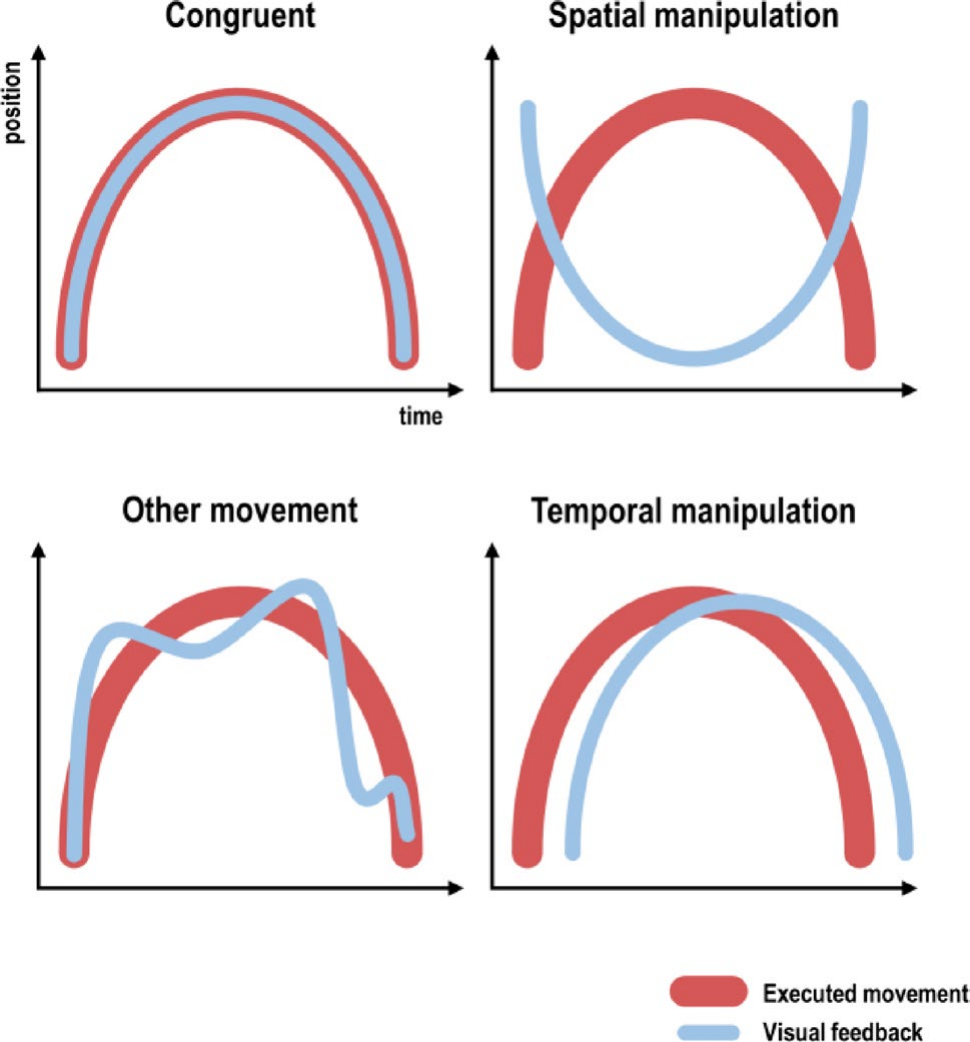
Common manipulations of (continuous) visual movement feedback for studying visuomotor self-other distinction. Shown is a schematic movement, where position changes along a single dimension, e.g. vertical displacement, with congruent feedback (top left); with spatially manipulated visual feedback (in this case, mirrored, top right); overlaid with another movement (bottom left); or with an added time delay (i.e., lagging, bottom right)

Visual movement feedback can be manipulated to be incongruent with executed movements in several ways: One can show the participant an entirely different visual movement (e.g., the experimenter’s), or one can displace (introducing spatial incongruence) or delay (introducing temporal incongruence) the visual movement with respect to the participant’s actual movement. Figure 3 shows examples of each kind of manipulation during a prototypical movement with continuous visual feedback (i.e., as during natural body movements). In principle, the same ideas apply to discrete movement feedback in other experimental settings; however, it should be noted that (some) kinematics of visual movements might need to be inferred from discrete feedback, which would introduce another computational step.

The idea to display another person’s movements to a participant during execution dates back at least to Nielsen’s (1963) “alien hand” experiment: His participants drew a line, while in some conditions seeing their own hand – in other conditions, they saw an image of the experimenter’s hand, which occasionally made deviating line drawings. This principle has been adopted by many subsequent studies; which displayed different gestures or movements of different fingers during the participants’ movements (e.g., Brass et al. 2009; Iacoboni et al. 2001; Kontaris et al. 2009; David et al. 2007). With the accessibility of MR compatible virtual reality technology, it is even possible to gradually morph self-generated movement signals with those of others, which opens up further interesting questions about the individual thresholds of visuomotor self-other distinction (see Yomogida et al. 2010; Nahab et al. 2011; Ohata et al. 2020; for examples). The common principle of studies using this kind of manipulation is that another person’s movements cannot be predicted (to the same extent) by one’s motor system; the observed movement should, therefore, result in an error response at the comparator that can be used for self-other distinction. In contrast, distorted visual movement feedback can be created from the participant’s own movements; i.e., by shifting them in space or time. For instance, spatial displacement can be implemented via mirrors or prisms; temporal lag can be implemented by introducing delays to video recordings of the participant’s movements or, more recently, to motion-captured movement data displayed through virtual hand models (Limanowski et al. 2017; Tanaka and Imamizu 2025).

An excellent overview of visuomotor mismatch processing can be found in the reviews and meta-analyses by (Jeannerod 2003; David et al. 2008; Sperduti et al. 2011; Archambault et al. 2015; Zito et al. 2020; Seghezzi et al. 2021). To summarize those: Most studies reported extensive brain responses to mismatching visual feedback including two or more of the candidate comparator regions (Fig. 2), among many others. Regions in and around the AG (with peak locations sometimes in the neighboring supramarginal gyrus, SMG) have been relatively consistently associated with detecting and processing visuomotor mismatches; including other movement-overlays (Farrer et al. 2008a, b; Nahab et al. 2011; Ohata et al. 2020) and delays (van Kemenade et al. 2017; Kemenade et al. 2019; Balslev et al. 2006; Limanowski et al. 2017; Vigh and Limanowski 2025). However, practically all of these studies have also reported qualitatively similar activation differences in the LOTC (i.e., including putative EBA regions) and/or the STS. Some studies have specifically highlighted the STS (Leube et al. 2003; using delays; Kontaris et al. 2009; using other movement-overlays), whereas others have emphasized the EBA (David et al. 2007, 2008; Yomogida et al. 2010; using other movements), or their interplay in processing different kinds of visual mismatches (Limanowski et al. 2017; using delays).

In sum, regarding the candidate visuomotor comparators shown in Fig. 2, the results are heterogeneous. The differences in experimental design between studies likely contributed to this. In particular, differences in the kind of executed movement and the degree of experimental control over it are particularly problematic when trying to disentangle visuomotor form visuo-proprioceptive comparisons. As shown schematically in Fig. 3, each manipulation of visual movement feedback (spatial, temporal, or overlay with another movement) implies a positional mismatch between executed and seen movements and, consequently, a mismatch of seen and felt body part positions. This means that, independently of the kind of visual manipulation, visual (e.g., EBA and STS, and potentially, AG) brain responses to the incongruence could be (partly or entirely) due to processing ‘static’ visuo-proprioceptive mismatches; i.e., frame-to-frame positional mismatches between seen and felt body positions.

The functional distinction between the candidate comparator areas could be improved by a systematic comparison of the errors (and their dynamics) resulting from the different kinds of feedback manipulation; especially spatial vs. temporal manipulations (Box 1, cf. Peters et al. 2025). Partly based on the similar empirical (neuronal and behavioral) responses to spatial and temporal visuomotor mismatches, some authors have proposed their principled comparability regarding self-other distinction (Farrer et al. 2008b; Krugwasser et al. 2019). In contrast, Rohde and Ernst (2016) have, convincingly, argued that sensory feedback delays are a different kind of error signal than spatial errors (displacement), because they signal changes to action timing and initiation; which links them much more closely to (violations of) volition and agency (see also Tanaka and Imamizu 2025; for a similar claim).

Relating to the functional specialization of the visual (biological) motion processing areas EBA and STS, one key distinction is that a constant spatial manipulation preserves the higher-order visual kinematics; i.e., the visual movement speed, acceleration, jerk, etc. always correspond to those of the actually executed movement. So, even though the moving body part’s position in space, or its posture, may be altered (mirror flipped, for instance), the participant still controls the visual kinematics “on-line”; i.e., as during normal movements with veridical feedback. In contrast, a time delay will alter all visual kinematics; i.e., the visual (body part) position in space *as well as* its speed, acceleration, jerk etc. For instance, speeding up one’s movement will not immediately result in an equally sped-up visual movement—vision will speed up only after the added delay, at a time when the executed movement speed may again have changed (exceptions are e.g. circular or sinusoidal movements with constant velocity, see Rohde and Ernst 2016). This has implications for how predictable the visual mismatches are initially. Thus, the “frame-by-frame” visual mismatch is harder to predict for delayed visual feedback, because the altered kinematics imply a nonlinear spatial transformation of position. In other words, the limb position and posture of delayed visual movements will not be predictable through an “easy” spatial transformation, as for displaced movement feedback. Therefore, one could expect larger error signals in the comparator(s) when processing delayed, compared with displaced visual movement feedback. An overlay with another visual movement entirely will potentially amplify this error response even further, as the visual mismatch over time is even less predictable. In the following section, I shall review approaches that try to capitalize on this kind of predictability; i.e., the fact that (some) errors can be learned by the motor system.

### Prediction and learning of new visual movement feedback: adaptation studies

A fundamental characteristic that distinguishes spatial *and* temporal distortions from overlays with entirely other movements (see above) is that the former two are, in principle, still predictable from one’s motor signals. This means that the brain’s internal models can re-learn visuomotor associations and feedback control: Through an adaptation of the forward model’s predictions by sensory prediction errors (from the comparison of predicted and actual feedback, see Fig. 1A), one can learn to move under the novel visual movement feedback (Shadmehr et al. 2010; Krakauer and Mazzoni 2011; cf. Ogawa et al. 2006, 2007; Grafton et al. 2008).

While a systematic analysis of temporal vs. spatial adaptation is missing, visuomotor adaptation studies have highlighted the role of regions in the LOTC (i.e., potentially including the EBA) during the early adaptation phase (reviewed in Limanowski, 2022). The PPC is, among other regions, also implied in this learning; however, visuomotor adaptation seems to predominantly engage the superior parietal lobule. This could indicate a selectively augmented processing of novel visual movement feedback, likely in the dorsal network, for more efficient learning. Thus, the STS or AG seem not particularly involved in processing vision for adaptation itself.

Using continuous hand-target tracking tasks with variably delayed virtual hand movements, we could show that the SMG (near the AG) and the STS responded parametrically and continually to the amount of visuomotor mismatch, while the LOTC (EBA) responded momentarily more strongly when novel visuomotor relationships were introduced (Limanowski et al. 2017; Vigh and Limanowski 2025). This could hint at an “earlier” role of the EBA in processing unpredicted visual feedback. Furthermore, we found that the SMG’s responses to delays were slightly attenuated during adaptation, while cerebellar signals were elevated (Vigh and Limanowski 2025). The cerebellar response profile is what one would expect from forward models (i.e., increased activity during delay suggesting computation of new predictions; cf. Kufer et al. 2024; Tzvi et al. 2022; Kilteni and Ehrsson 2024), whereas the attenuation in the SMG is what one would expect of a “comparator” (i.e., reduced error signal after adaptation, because the visual feedback is now better predicted). Thus, in principle, adaptation designs could contribute to isolating visuomotor comparisons. However, as visuomotor adaptation may entail visuo-proprioceptive recalibration (Henriques and Cressmann, 2012; Block et al. 2013; Limanowski, 2022; Kufer et al. 2024), lower ‘mismatch responses’ following adaptation could also result from recalibrated sensation rather than adapted forward predictions.

Therefore, it is worth investigating differences between spatial vs. temporal manipulations of visual movement feedback; i.e., the corresponding adaptation process. Recall that spatial manipulations preserve the timing of visual kinematics (see above). This means that any visually conveyed movement error (i.e., a mismatch between desired and actual visual movement consequences) can be used to adjust control just as during ‘normal’ action—requiring only an additional transformation of (visual) coordinates in space. This allows for efficient feedback control and visuomotor adaptation. However, based on the assumption that in feedback control systems like the brain’s, the forward model entails a prediction of sensory delays (potentially, a “Smith predictor”, Foulkes and Miall 2000; Miall et al. 1993; see above), the delay predictions of internal models could be re-learned as well. Thus, although delay adaptation has received comparably less attention from researchers than spatial adaptation (but see the excellent synthesis by Rohde and Ernst 2016), it has received some experimental support (Foulkes and Miall 2000; Botzer and Karniel 2013; Perrinet et al. 2014). Correspondingly, Wen (2019) concluded that participants may indeed experience control during visual delays. It should, however, be noted that delay adaptation likely has limits—particularly for goal-directed movements with complex movement trajectories (Perrinet et al. 2014). The main reason for this is that the time between the actual execution of an “error” and a potential error correction, i.e., a correction of the motor commands through adjusted forward sensory predictions, increases with the delay. In other words, error correction will become increasingly more “off-line” under large delays, which renders feedback control much more ineffective or even impossible (Foulkes and Miall 2000; Leib et al. 2024).

The key difference to spatial displacements is that for delay adaptation, the delay component of the forward model, not its predictions of position, needs updating. Delays could hence be a more domain general ‘agency’ error signal (Rohde and Ernst 2016). A resulting empirical (neuroanatomical) question is whether sensory feedback delays are also registered by different comparators than spatial manipulations—and whether appropriately designed studies could, thusly, contribute to identifying the loci of visuomotor comparators (Box 1).

#### Manipulations of visual movement feedback during passive vs active movements

Brain responses to visuomotor self-other distinction can be isolated through the combined (factorial) manipulation of visual feedback congruence and the locus of movement generation; i.e., active, self-generated vs. passive, externally generated. Passive (externally induced) body movements of the participant generate identical visual and proprioceptive feedback as active movements, but lack the respective motor predictions. The resulting interaction effect should isolate brain responses specifically related to the visuomotor comparison process: A brain region responding more strongly (or even exclusively) to mismatching visual movement feedback during active, compared with passive movements (Fig. 1A). A similar logic has been applied in the somatosensory domain, starting with the seminal experiments by Blakemore et al. (1998; cf. Weiskrantz et al. 1971). However, only a handful of brain imaging studies have used such “interaction” designs in the visuomotor domain:

Christensen and colleagues (2007a*)* compared active (self-generated) vs. passive ankle movements, with or without visual movement feedback conveyed by a cursor drawing a curve reflecting a goniometer signal from the foot. The passive movements were implemented by an experimenter moving the foot. Passive > active movements were associated with increased BOLD signal in the bilateral TPJ (including regions around the AG and STS), alongside many other brain areas, including the medial and superior frontal cortex and bilateral sensorimotor cortices. The interaction effect – looking at stronger activation increases to visual feedback > no vision during passive relative to active movements – revealed significant effects near the bilateral STS, the anterior temporal cortex, and medial, superior, and inferior frontal cortices.

Taking an analogous approach in fMRI study, Limanowski et al. (2018) had participants perform right-hand rotations actively, or those rotations were generated by the experimenter (i.e., passive movements); in each case with or without visual feedback of the moving hand. Here, the movement feedback was conveyed by a photorealistic 3D virtual hand model via motion-tracking. Passive, compared with active movements overall increased activity in the bilateral STS (alongside further activations in parietal and superior frontal cortices). There was a significant interaction effect between visual feedback and agency in the right posterior STS: its response to visual movement feedback > no feedback was significantly increased during passive, compared with active movements. This suggested that the right STS evaluated visuo-motor mismatches.

Other studies have added temporal delays to the visual action feedback. In an fMRI study by Balslev and colleagues (2006*)*, participants moved a sliding mouse with their finger, or the experimenter moved their finger analogously; while participants viewed movement feedback via a cursor, which moved synchronously or asynchronously (here, the viewed movement was a play-back of previous trials, which was either leading or lagging the actual movement). FMRI scanning revealed increased activation in the right AG by asynchronous compared with synchronous feedback during both active and passive movements. There was no significant interaction effect; i.e., no differential processing of incongruence depending on agency. The authors concluded that the observed AG activation was, therefore, indicative of visuo-proprioceptive, rather than visuo-motor comparisons.

In a study by Tsakiris and colleagues (2010*)*, participants lifter their index finger – or the experimenter lifted it – while viewing camera captured videos of the finger movement; which could be synchronous (with an intrinsic delay of 100 ms) or asynchronous (delayed by 500 ms). The fMRI analysis showed several clusters of activation related to the main effect of asynchronous > synchronous visual feedback, which included bilateral temporo-parietal regions around the AG. Furthermore, the main effect of passive > active movements was reflected by relatively stronger activation of several brain areas, including the bilateral STS alongside medial and superior frontal areas, the precuneus, the cingulate gyrus, and the bilateral postcentral gyrus. No interaction effects were reported. Based on the slightly (but non-significantly) different activation profiles of several regions around the right TPJ, the authors speculated that some of them (around the AG) may register visuo-proprioceptive conflicts, while others, in the more superior SMG, may play a role in visuomotor comparisons and agency attribution.

Another group of researchers has used elegant pneumatic setups allowing the generation of passive (vs. active) hand movements inside the MR scanner, to investigate the agency dependent processing of visual movement feedback. Thus, in van Kemenade et al. (2019), participants either performed active hand movements, or passive hand movements were executed via the pneumatic device; while participants viewed camera recordings of their hand, which were delayed by variable amounts. In this study, the authors focused their analysis on BOLD signal correlations with the amount of delay in each condition; i.e., on the interaction effect of agency with delay. The respective main effects (differences between conditions) were subsequently reported in Arikan et al. (2019): Passive > active movements were, on average, associated with stronger activity in a wide spread network of brain areas. Thereby, the strongest effects and largest clusters obtained from this contrast were located in the bilateral STS. Similarly, the amount of visual delay was positively correlated with activity in the right LOC and the bilateral STS; however, without significant differences between active and passive conditions in these areas (such a difference was found in the right cerebellum). Based on the absence of an interaction effect (i.e., of differences in delay correlation between active and passive movement conditions), the authors concluded that activity in these areas more likely reflects general visuo-proprioceptive conflict detection than an agency specific i.e., visuomotor process. In a variation of this design, Uhlmann et al. (2020) looked at active vs. passive movements (under delayed visual feedback) while also manipulating the visual hand identity to be “self” or “other”. The fMRI results revealed an overall increased activity in the bilateral STS, alongside further frontal, parietal, and cerebellar activations, during passive > active trials; and, moreover, stronger STS and hippocampal activity during trials in which the seen hand identity was “other” > “self”. An interaction effect was found in the bilateral AG, the bilateral superior frontal cortex, the precuneus, and the middle temporal gyrus; i.e., in these regions, activity was increased during passive > active movements when one’s own hand > someone else’s hand was seen. This suggests that forward models may incorporate visual hand identity into their predictions.

In sum, the anticipated interaction effect was not coherently observed in the reviewed studies. The only coherent finding across the reviewed studies was the increased STS (and, in some studies, AG) activation by observed visual movement feedback generated by passive, compared with active movements. This result is consistent with work in monkeys (Hietanen and Perrett 1996) and with the previous observation that the STS responds more strongly to observation of others’ movements (see above). Activity increases in the IPL/STS can also be found in passive > active movements without any visual feedback (Limanowski et al. 2020; cf. Jaeger et al. 2014); which could indicate such STS responses reflecting differences in “passivity” (task disengagement). Possibly, the STS responses could also encode social aspects of the experiment such as interactions with the experimenter guiding the passive movement (but several studies found these responses using automated induction of passive movements, e.g., Uhlmann et al. 2020; Limanowski et al. 2020).

Nevertheless, these kinds of interaction designs should be pursued to isolate visuomotor from visuoproprioceptive comparisons in the brain. One potential reason for the inconsistent effects in the above studies are methodological problems associated with the very challenging implementation of “truly passive” movements. Pushing or pulling body parts generates all kinds of somatosensation (on the skin, in the joints etc.). Participants might notice this especially at the movement onset, and move “cooperatively”. Future work could explore the use of functional electrical muscle stimulation to induce passive movements; i.e., through stimulation of the same muscles (cf. Iftime-Nielsen et al. 2012; Limanowski et al. 2020). Another potential modification of the above designs is depriving the participants of proprioceptive feedback; which can be done by using ischemic nerve block or anesthetics (e.g., Christensen et al. 2007b). The absence of proprioceptive information would make an interpretation of brain activity increases during incongruent visual movements in terms of mere visuo-proprioceptive comparison unlikely. These experiments could be linked to neuropsychological findings in deafferented (i.e., with a partial or complete lack of proprioceptive afferent) patients; i.e., these patients can adapt to distorted visual movement feedback, but may be unaware of the underlying learning and recalibration process (e.g., Fourneret et al. 2002; Farrer et al. 2003a, b; Miall and Cole 2007). While there are still many controversies about what kind of afferent signals can be received by these patients (Miall and Cole 2007), neuropsychological case studies open up a unique perspective on which comparison processes are truly fundamental for motor control and the conscious access to the underlying computations (cf. Jeannerod 1986; Wolpert et al. 1998; Sirigu et al. 1999). Finally, the induction of passive (vs. active) movements can be combined with visuomotor adaptation tasks. From a computational perspective, which focuses on learning through updates of the motor system’s forward model (see above), action should be necessary for adaptation. Indeed, there is substantial evidence that sensorimotor adaptation is impaired during passive movements (Lackner 1977; cf. Henriques and Cressmann, 2012; Rohde and Ernst 2016; Kufer et al. 2024). Thus, any significant interaction effects in the cortical adaptation-related activity and connectivity could, in principle, be interpreted as indicating visuomotor comparisons during action, specifically.

## Conclusion

In the past years, many important insights have been gained into the neurocomputational architecture underlying visuomotor self-other distinction. Thereby the forward/comparator model framework has proven valuable for hypothesis generation and for the design of brain imaging studies testing these hypotheses. There is considerable support for an implication of the AG, EBA, and STS in self-other distinction based on visual and action cues. While the question whether the “action cues” received by those regions are motor or non-motor by nature cannot yet conclusively be answered (among other questions, Box 1), there are promising methodological developments suggesting that, in principle, it can be. 

Box 1: key open questions

- Where is the visuomotor comparator – if there is a single one?
  Some of the reviewed evidence suggests supramodal comparisons in the AG, while other studies suggest specific comparisons in the EBA or the STS; i.e., regions specialized in visual body/motion perception. Models of biological motion processing or social cognition imply a serial communication of the EBA and the STS (e.g. Giese and Poggio 2003), EBA and the AG (Jeannerod 2004), and of the AG/TPJ and the STS (e.g. Patel et al. 2019). This could be reconciled within a hierarchical process of self-other distinction, in which these regions communicate with each other. An interesting question is whether there are specialized comparator “modules” or, rather, gradients (cf. Beauchamp et al. 2002; Lingnau and Downing 2015) in self-other distinction as well.
- Do ventral visual areas receive motor signals—and do they implement comparators or forward models?
  Visual processing in the EBA or STS may be biased by action cues, but whether these regions receive motor signals such as the efference copy has not yet been demonstrated clearly enough. Functional and structural connectivity studies in humans should identify potential afferents from motor regions; e.g., those supposedly implementing forward models. Furthermore, regions in the LOTC encode parameters of upcoming actions, which could imply a role closer to a forward model (Gallivan and Culham, 2015; cf. Zimmermann et al. 2016, 2018; Quirmbach and Limanowski 2024). Ventral visual areas could thus themselves compute the forward predictions based on motor signals, rather than receiving those predictions and comparing them against sensory reafference. This could (but need not) mean that visuomotor evaluations are reserved for other brain areas like the cerebellum or the PPC (cf. Blakemore et al. 1998; MacDonald and Paus 2003).
- Does hemodynamic activity in comparator modules (only) reflect mismatches?
  The common assumption reviewed here is that comparators can be identified through their increased (hemodynamic) responses to mismatching action feedback, reflecting population responses to sensorimotor prediction errors evoked by unpredicted movement feedback. However, “sharpening” accounts propose a selective augmentation of neuronal responses tuned to predicted (self-generated) action feedback (Yon et al. 2018). This could mean that comparators show more complex, contextual activity profiles; and that experiments should account for this (Press et al. 2020).
- What is the contribution of spatial vs temporal cues to visually based self-other distinction?
  Different kinds of visual distortions imply differences in kinematic predictability and, potentially, control and learning—all of which could influence visuomotor evaluations in the candidate comparator areas. A comparison of the sensitivity to, and the—potentially separate or hierarchical—processing of spatial vs temporal distortions is necessary. This can be addressed by designs systematically combining more than kind of manipulation in appropriate time-resolved designs.
- How can we achieve better experimental control over non-motoric cues?
  Purely sensory cues, such as proprioception and its congruence with vision, can also be used to ascribe observed movements to oneself or another. To isolate motor comparators, new methods of generating truly passive movements via e.g. muscle stimulation are needed. These could be combined with adaptation designs, which offer another angle at locating motor predictions; or with the addition of noise or a temporary attenuation of proprioception. This research would contribute to answering whether or not there are sensorimotor comparators that ignore intersensory congruence.

## Data Availability

No datasets were generated or analysed during the current study.

## Footnotes

1 In this review, I stick with the labels “EBA”, “AG”, and “STS” for consistency with previous proposals (Jeannerod 2004; David et al. 2007). It should be mentioned that not all of the reviewed studies functionally localized the EBA; hence a broader label would be “lateral occipitotemporal cortex” (Lingnau & Downing, 2016). Similarly, I consider reported hemodynamic activations with peaks in or around the AG or STS; i.e., also discussing those in the respectively neighboring SMG or superior temporal gyrus.
